# Association Between Hypomineralisation of Second Primary Molars and Tooth Agenesis

**DOI:** 10.1111/ipd.70148

**Published:** 2026-09-23

**Authors:** Débora Juliana de Araújo Lopes, Eloisa Cesario Fernandes, Jaderson Felipe Santos Dantas, Patrícia Bittencourt Santos

**Affiliations:** ^1^ Dental School State University of Rio Grande do Norte ‐ UERN Caicó Rio Grande do Norte Brazil; ^2^ Department of Dentistry Federal University of Rio Grande do Norte ‐ UFRN Natal Rio Grande do Norte Brazil; ^3^ Department of Paediatric Dentistry Federal University of Rio Grande do Norte ‐ UFRN Natal Rio Grande do Norte Brazil

**Keywords:** dental enamel hypomineralisation, paediatric dentistry, primary tooth, tooth abnormalities

## Abstract

**Background:**

Hypomineralisation of second primary molars (HSPM) is an enamel defect with a multifactorial and potentially genetic aetiology. While the association between enamel defects and dental anomalies is a relatively recent area of investigation, it has not yet been explored specifically in cases of HSPM.

**Aim:**

To assess the association between HSPM and tooth agenesis (TA).

**Design:**

A retrospective cross‐sectional study was performed using pre‐treatment records of patients aged 7–12 years. A total of 285 patient records were evaluated. Statistical analyses were performed using the Fisher's exact test and logistic regressions.

**Results:**

A higher prevalence of TA was observed among children with HSPM‐affected teeth (95% CI: 1.07–3.17, *φ*‐coefficient: 0.238, PR: 1.84). Individuals with severe HSPM had significantly greater odds of TA (OR = 12.47; *p* < 0.001; 95% CI: 3.47–24.70). Patients with HSPM had a significantly higher likelihood of experiencing TA (OR = 6.97), particularly second premolar agenesis (OR = 11.80; *p* < 0.001; 95% CI: 2.83–49.10).

**Conclusion:**

An association was found between HSPM and TA, particularly involving mandibular second premolars. Moreover, increased HSPM severity was linked to a higher risk of developing TA.

## Why This Paper Is Important to Pediatric Dentists

- Patients with HSPM present a higher prevalence of tooth agenesis, particularly of mandibular second premolars.
- Individuals with severely affected molars had higher odds of tooth agenesis.
- This paper supports the hypothesis that HSPM and tooth agenesis may share common genetic origins.

## 1 Introduction

Hypomineralised Second Primary Molar (HSPM) is a qualitative enamel defect manifesting as demarcated enamel, affecting at least 1 s primary molar and often involving primary canines [1]. Hypomineralised enamel appears as an opacity with distinct borders which can present in shades of white, yellow, or brown and may occur either with or without enamel destruction due to post‐eruptive breakdown (PEB) from occlusal forces [1, 2]. Compared to healthy teeth, HSPM‐affected enamel presents a less distinct prism sheath, reduced hydroxyapatite content, and diminished mechanical properties, such as hardness and elasticity [3]. A systematic review highlighted a worldwide HSPM prevalence of 6.80% with variations observed across different countries [4].

The aetiology of HSPM appears to be multifactorial but remains inconclusive [5]. Several risk factors have been suggested, including prenatal conditions such as maternal hypertension and smoking during pregnancy [6]. Perinatal factors like low birth weight, delivery complications, prematurity, and need for incubator care have also been associated with HSPM. In addition, postnatal factors such as antibiotic use, asthma, and fever episodes have frequently been implicated [5].

Recently, an association between HSPM and genetic variants leading to the retention of organic matrix proteins, resulting in a subsequent decrease in the mineral content of tooth enamel, has also been proposed [7, 8]. Indeed, a systematic review has revealed that genetic mutations could potentially arise during dental enamel formation, leading to disruptions in the maturation stages of enamel [7]. On the other hand, odontogenesis, as well as amelogenesis, is under strict genetic control, and the shade, shape, mineralisation, and the presence of tooth germ can all be affected by genetic variation [9]. Many studies have demonstrated a strong genetic influence on tooth agenesis (TA). Twin and family studies have determined that some tooth absences are inherited via an autosomal dominant pattern, with incomplete penetrance and variable expressivity [10].

The potential for other dental anomalies to occur in children with enamel hypomineralisation has only recently gained attention. A British hospital‐based study reported a higher prevalence of TA among patients with molar incisor hypomineralisation (MIH) compared to the general population [11]. Following this, several studies have investigated the association between MIH and TA, although the findings have been inconsistent. More recently, a cross‐sectional study conducted across 14 countries reported a higher prevalence of hypodontia in individuals with MIH compared to those without the condition. While the association between MIH and dental anomalies has been explored, no studies to date have investigated this relationship in cases of HSPM. Therefore, this study aimed to assess the association between HSPM and TA, under the hypothesis that HSPM acts as a risk factor for tooth absence.

## 2 Materials and Methods

### 2.1 Ethical Aspects

This study was approved by the Research Ethics Committee of the State University of Rio Grande do Norte (no. 4.708.279) and adhered to the guidelines outlined by the Council for International Organisations of Medical Sciences concerning the use of clinical records for research. Written informed consent was obtained for the publication of the images.

### 2.2 Study Design, Participants, and Setting

This retrospective cross‐sectional survey is reported following the STROBE (Strengthening the Reporting of Observational Studies in Epidemiology) statement.

Sample size calculations were conducted using G*Power Version 3.1.9.7 to ensure suitable proportions and comparisons. Based on a pilot study involving 70 patients with a confidence level of 95% and a power of 80%, a minimum sample size of *N* = 251 was determined for conducting a cross‐sectional study on TA. The expected prevalence rates of TA found in patients with and without HSPM were 12.1% and 5.1%, respectively.

This retrospective cross‐sectional study analysed digital pre‐treatment records, including panoramic radiographs, intraoral photographs, and medical history of non‐syndromic patients who sought dental care at the Federal University of Rio Grande do Norte voluntarily, accompanied by their parents or guardians. Data were analysed between June 2023 and January 2025.

All patients aged 7–12 years with four erupted second primary molars were included. Syndromic patients and those with dental fluorosis, enamel hypoplasia, or amelogenesis imperfecta were excluded. Individuals with blurred, dark, pixelated radiographs/photographs or when it was not possible to visualise all tooth surfaces were also excluded.

### 2.3 Variables

The study focused on TA as the primary outcome variable. The presence of HSPM was considered the exposure of interest.

### 2.4 Data Collection

#### 2.4.1 HSPM

Second primary molars were diagnosed with HSPM based on the criteria established by Elfrink et al. [2]. They are as follows: white‐yellow‐brown demarcated opacities, PEB associated with opacities, atypical extensive caries with surrounding opacities, atypical restorations of a size and location unrelated to the caries pattern, and extractions due to HSPM (Figures 1 and 2).

**FIGURE 1:**
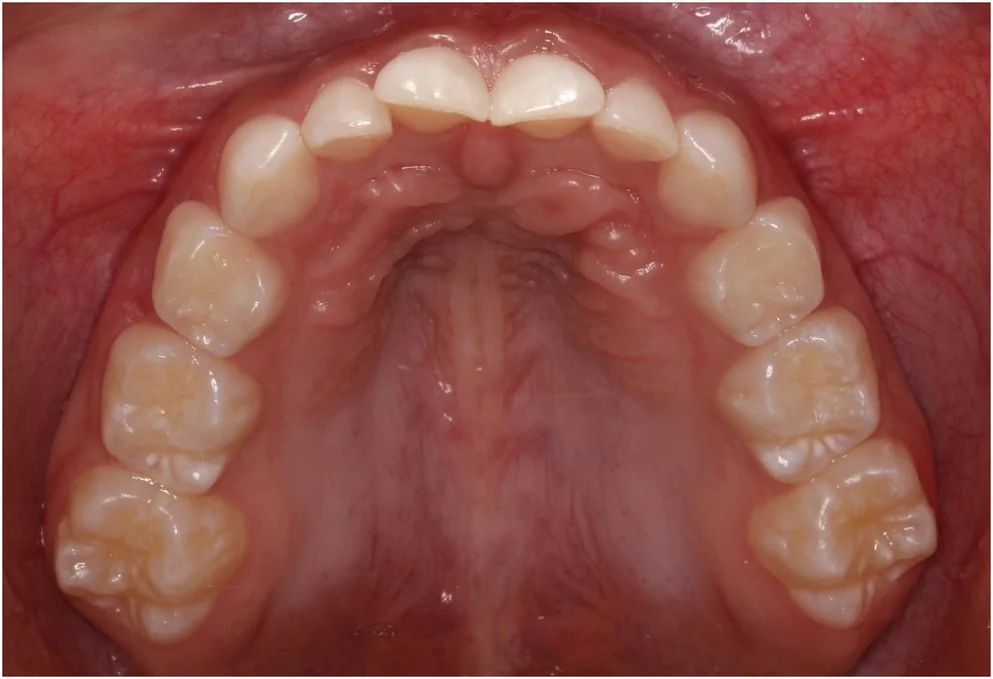
Opacities without PEB.

**FIGURE 2:**
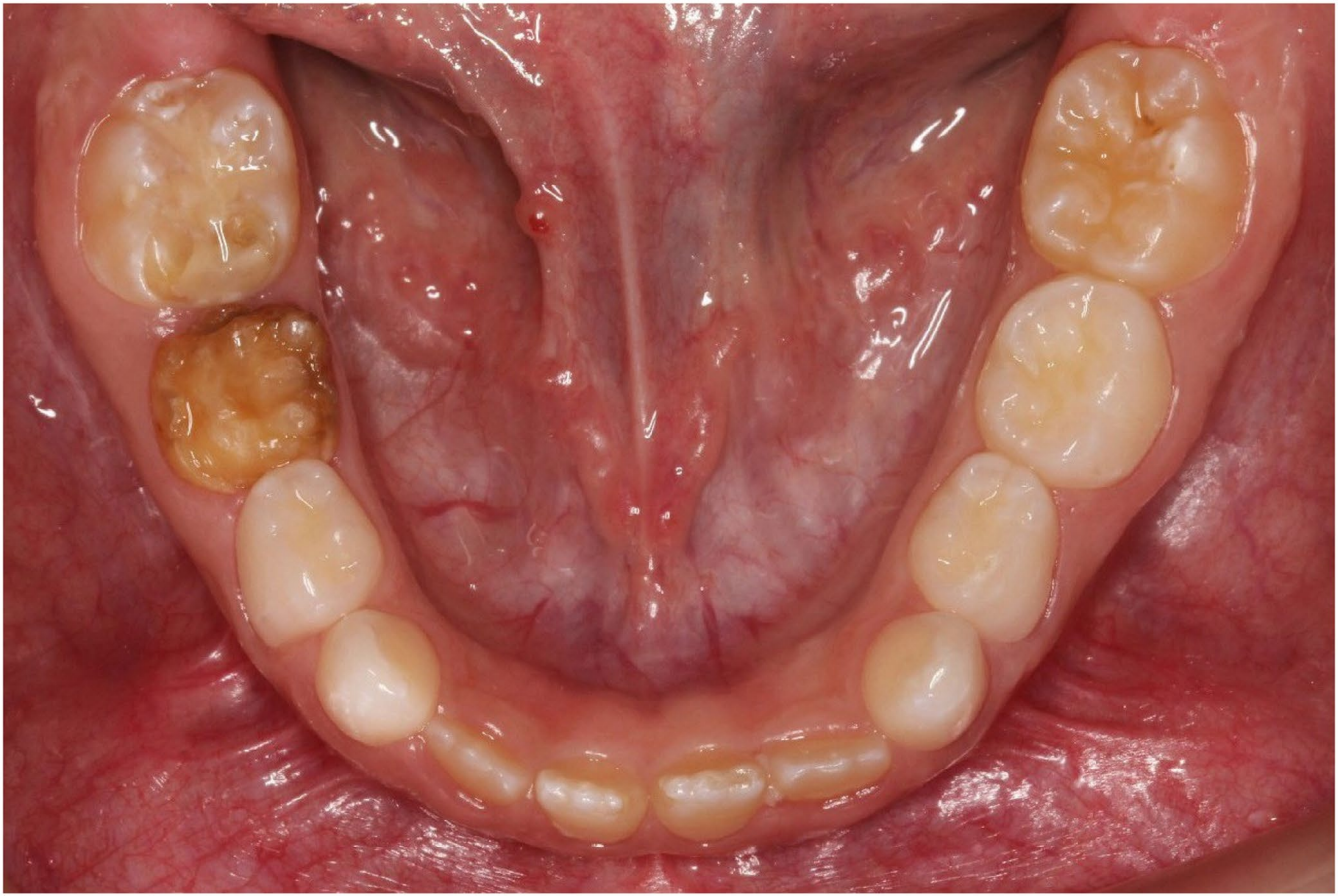
Post‐eruptive breakdown.

#### 2.4.2 Bias

Training and calibration sessions were performed. The initial stage involved establishing standardised diagnostic criteria for HSPM. The calibration of the examiner followed the protocol suggested by Ghanim et al. [12] and the validity of these photographs was established by Elfrink et al. [13]. These were meticulously reviewed and discussed by a qualified and experienced paediatric dentist who was considered the gold standard.

For the second phase, a set of 40 clinical records was utilised to assess the varying degrees of hypomineralisation and to consider potential alternative diagnoses, including fluorosis, enamel hypoplasia, and amelogenesis imperfecta. The records were re‐examined during the study, and the intra‐examiner reproducibility was excellent (Cohen's Kappa coefficient = 0.92).

Tooth agenesis was defined as the congenital absence of the tooth germ. The same examiner reviewed all radiographs using a uniform protocol (Cohen's Kappa coefficient = 1.00). Inter‐examiner agreements were 0.94 for HSPM and 1.00 for TA.

Digital photographs were obtained using a Canon EOS Rebel T1i camera, ring flash, and 100‐mm macro lens (all Canon, Tokyo, Japan) at a standardised 1:1.2 magnification and manual parameters (ISO 200, T: 1/200, F: 25). These photographs were taken by a single operator following standard clinical procedures and were digitally processed using Adobe Photoshop Lightroom Classic CC, version 12.4, by Adobe Inc. During data collection, assessments were carried out in a semi‐darkroom environment using a desktop computer with a 21.5‐in. screen (1920 × 1080 pixels/in.) and preview software, version 11.0 for macOS, operating in full‐screen mode.

### 2.5 Statistical Analysis

Descriptive statistics were performed, and data were analysed for frequency, sex, age, and type of tooth involved. The association between TA and the presence of HSPM was assessed using Pearson's Chi‐square test, Fisher's exact test, and Student's *t*‐test, as appropriate. Additionally, binary logistic regression analysis was conducted to further evaluate the association between HSPM severity and TA. Results were presented with prevalence ratios (PR) or odds ratios (OR), 95% confidence intervals (CI), and corresponding *p*‐values.

## 3 Results

### 3.1 Participants and Descriptive Data

Initially, 1387 pre‐treatment records were evaluated, from which the following were excluded: 601 due to the natural exfoliation of primary second molars (expected for this evaluated age), 341 due to the absence of a panoramic radiograph, 73 due to poor record quality, and 87 due to difficulties in examining tooth surfaces. The final sample consisted of 285 patients, 126 of whom were males. The participants had an average age of 8.77 ± 1.68 years. The prevalence of HSPM was 16.1%. HSPM was significantly more prevalent in males than females (22.2% vs. 11.3%, *p* = 0.013). The profile of the participants is detailed in Table 1.

**TABLE 1:** Characteristics of the study patients.

	HSPM *N* (%)		*p*
	Yes	No	
Sex			
Female	18 (11.3)	141 (88.7)	0.013^#^
Male	28 (22.2)	98 (77.8)	
Severity			
Mild	29 (63.04)		
Severe	17 (36.95)		
Mean (SD)			
Age	8.72 (1.60)	8.77 (1.70)	0.851*

^#^ Pearson's chi‐squared test.

* Student's *t*‐test.

### 3.2 Main Results

Table 2 presents the association between HSPM and TA. A higher prevalence of TA was observed among children with HSPM than among those without HSPM (PR = 1.84; 95% CI: 1.07–3.17; *φ* = 0.238). Children with HSPM also had a 2.57‐fold higher prevalence of mandibular second premolar agenesis than those without HSPM. No significant association was found between HSPM and maxillary premolar or lateral incisor agenesis.

**TABLE 2:** Association between tooth agenesis and HSPM.

	HSPM		*p* *	PR	CI 95%	*φ*‐coefficient
	No *N* (%)	Yes *N* (%)				
TA						
Absent	232 (85.9)	38 (14.1)	< 0.001	1	1.07–3.17	0.238
Present	7 (46.7)	8 (53.3)		1.84		
Maxillary Premolar						
Absent	236 (84.0)	45 (16.0)	0.507	1	0.63–1.98	0.028
Present	3 (75.0)	1 (25.0)		1.12		
Mandibular Premolar						
Absent	236 (85.5)	40 (14.5)	< 0.001	1	1.02–6.47	0.248
Present	3 (33.3)	6 (66.7)		2.57		
Maxillary lateral Incisor						
Absent	236 (84.3)	44 (15.7)	0.185	1	0.68–2.88	0.086
Present	3 (60.0)	2 (40.0)		1.40		

* Fisher's exact test.

Table 3 shows the association between HSPM severity and TA. Logistic regression analysis revealed that HSPM severity was significantly associated with TA. Patients with mild HSPM did not have significantly higher odds of TA than those without HSPM (*p* = 0.063; 95% CI: 0.79–13.21). However, individuals with severe HSPM had significantly higher odds of TA (OR = 12.47; 95% CI: 3.47–24.70; *p* < 0.001).

**TABLE 3:** Association between TA and HSPM according to severity.

	OR	*p* *	95% CI
HSPM			
Absent	1		
Mild	3.82	0.063	0.79–13.21
Severe	12.47	< 0.001	3.47–24.70

*Note:* Nagelkerke's (*R* ^2^_N_) = 0.146.

Abbreviations: CI, confidence interval; HSPM, hypomineralisation of second primary molars; OR, Odds Ratio.

* Binary logistic regression.

Table 4 presents the logistic regression analysis. Patients with HSPM had significantly higher odds of tooth agenesis (TA) than those without HSPM (OR = 6.97; 95% CI: 2.39–20.35; *p* < 0.001). This association was particularly evident for mandibular second premolar agenesis (OR = 11.80; 95% CI: 2.83–49.10; *p* < 0.001).

**TABLE 4:** Logistic regression for TA as outcome.

Independent variable	Agenesis											
	Tooth			Maxillary premolar			Mandibular second premolar			Maxillary lateral incisor		
	OR	p*	CI 95%	OR	p*	CI 95%	OR	p*	CI 95%	OR	p*	CI 95%
HSPM												
Absent	1	< 0.001	2.39–20.35	1	0.632	0.17–17.1	1	< 0.001	2.83–49.10		0.170	0.58–22.02
Presence	6.97			1.74			11.80			3.57		
*R* ^2^_N_	0.120			0.005			0.169			0.035		

*Note:* Nagelkerke's (*R* ^2^_N_), VIF 1.01–1.01, Tolerance 0.95–0.95.

Abbreviations: CI, confidence interval; HSPM, hypomineralisation of second primary molars; OR, Odds Ratio.

## 4 Discussion

To the best of our knowledge, this is the first study to provide data regarding the correlation between HSPM and TA. Our results showed a higher prevalence of TA in children with HSPM‐affected teeth. Since odontogenesis is under genetic control, it is conceivable that genes may play a role in the onset of both dental anomalies [8]. Tooth development involves a sequential and reciprocal set of signalling interactions [14]. Disturbances at any stage of tooth development can affect growth, differentiation, and pattern formation [15]. Mutations in several genes have been reported as causal factors for TA, and epigenetic regulation affecting a network of interconnected signalling pathways related to tooth development has also been suggested as a factor in the aetiology of this dental anomaly [16]. While the aetiology of HSPM remains inconclusive, a recent study also has highlighted the interaction of systemic, epigenetic, and genetic factors [17]. The recognition of the likelihood of additional dental anomalies occurring in children with enamel defects has emerged relatively recently [18].

Tooth agenesis was observed in 17.39% of patients with HSPM. Comparisons with previous studies should be interpreted with caution, as no research has investigated dental anomalies in children with enamel defects in the primary dentition. However, recent studies have demonstrated a higher prevalence of TA in patients with MIH. In fact, individuals with MIH are reported to have a 1.5 times higher prevalence of TA compared to unaffected peers. Furthermore, when considering only patients with TA while excluding other associated dental anomalies, the risk of TA in MIH patients increased by 2.74 times [18]. Despite these findings, Marcianes et al. reported that the frequency of TA in MIH and non‐MIH groups was 7% and 8%, respectively, with no statistically significant difference [19]. Another study also found no significant difference in the overall prevalence of dental anomalies between children with and without MIH, although differences in the distribution patterns of specific dental anomalies were observed [20]. Given these inconsistencies, a large‐scale, multicentre study aimed to assess the prevalence of various dental anomalies in children with and without MIH. Researchers from 14 different countries collected clinical and radiographic data from 1279 patients. Their findings align with our study, as they reported that the adjusted odds of TA were 1.49 times higher in children with MIH compared to those without. These results support the role of (epi) genetic factors in the aetiology of both MIH and HSPM, suggesting that disturbances in tooth development likely result from a complex interplay of environmental, genetic, and epigenetic influences rather than a single causative factor [21].

Lower second premolars are the most commonly missing teeth, excluding third molars, and have been associated with other dental anomalies [22]. The first studies to identify patterns of associated dental anomalies were conducted by Garn et al. (1961) [23] and Garn and Lewis (1962) [24], who observed that individuals with mandibular second premolar agenesis exhibited a higher prevalence of additional dental anomalies [25]. Our findings reinforce this concept, strongly suggesting that both HSPM and TA are multifactorial genetic conditions. These findings suggest that multiple genes contribute to both anomalies, each exerting a small individual effect [26]. Furthermore, the high prevalence of second premolar agenesis raises questions about whether HSPM could directly impact its permanent successor. To clarify this relationship, a longitudinal prospective cohort study is recommended.

The relationship between TA and HSPM is influenced not only by prevalence but also by severity, indicating that individuals with more severe HSPM face a higher risk of developing this anomaly. A similar pattern has been observed in MIH, where the severity of lesions is linked to an increased likelihood of TA. This suggests that the association between HSPM and TA may mirror that seen in enamel defects affecting permanent teeth. This connection could be explained by the shared developmental timeline of second primary molars and first permanent molars. Both teeth undergo amelogenesis and mineralisation during overlapping periods, meaning that early‐life disturbances could simultaneously impact both dentitions. Therefore, children with severe HSPM, particularly those with a poor long‐term prognosis, require precise management due to the increased risk of second premolar agenesis. If not closely monitored, severe cases may lead to early extractions, and undiagnosed agenesis of the successor could compromise occlusion. Given the increased likelihood of TA in children with HSPM, a panoramic radiograph during the early mixed dentition stage (around 7 years) could be crucial for treatment planning. Early identification of missing teeth enables clinicians to provide timely and optimal care, especially in severe cases.

The prevalence of HSPM found was 16.1%, which falls within the wide range reported in other studies examining hypomineralisation in primary teeth [1, 27]. However, the prevalence in our study was higher compared to the values recently reported in the systematic review [4]. Comparing these studies remains challenging due to variations in diagnostic criteria, examination methodologies, differences in age groups, ethnicity, and a possible influence of environmental factors. This may explain the higher prevalence observed in less developed regions. Our results closely resonate with outcomes from other Brazilian study, where prevalence rates have reached as high as 20% [28]. These disparities might be linked to distinctions in socio‐economic conditions, healthcare accessibility, and various regional factors that have been associated with the development of HSPM.

The limitations of our study relate to the inclusion of patients older than the recommended age of 5 years for HSPM diagnosis [2]. In older children, enamel opacities may become less detectable due to prolonged exposure to masticatory forces and wear [6]. However, assessing very young children may lead to an overestimation of TA, particularly second premolar agenesis, due to potential delays in dental development. Additionally, this study is a retrospective analysis of routinely collected clinical service data. The data were not collected specifically for this study, which may lead to selection bias, especially given the exclusion of a significant amount of data. Furthermore, our sample was limited to patients from a single hospital, which may not fully represent the broader population.

In conclusion, there is an association between HSPM and TA, particularly for mandibular second premolars. Additionally, increased severity of HSPM is linked to a higher risk of TA. However, no association was found between HSPM and agenesis of maxillary premolars or lateral incisors.

## Author Contributions

We certify that we participate sufficiently in the design of the project and/or analysis of the data obtained and/or in the final writing of the article and that everyone agrees with the version sent for publication. **Débora Juliana de Araújo Lopes:** collected the data, analysed the data and led the writing; **Eloisa Cesario Fernandes:** collected the data, analysed the data and led the writing; **Jaderson Felipe Santos Dantas:** analysed the data and led the writing; **Patrícia Bittencourt Santos:** conceived the ideas, analysed the data and led the writing.

## Funding

One of the authors received a doctoral scholarship from the Coordination for the Improvement of Higher Education Personnel (CAPES), Brazil.

## Ethics Statement

This research was approved by the Research Ethics Committee of the State University of Rio Grande do Norte (no. 4.708.279).

## Conflicts of Interest

The authors declare no conflicts of interest.

## Data Availability

The data that support the findings of this study are available on request from the corresponding author. The data are not publicly available due to privacy or ethical restrictions.
